# Efficient generation of human induced pluripotent stem cells from urine samples of patients with Fragile X syndrome

**DOI:** 10.3389/fcell.2024.1489190

**Published:** 2024-11-22

**Authors:** Olivier Dionne, Salomé Sabatié, Fléchère Fortin, François Corbin, Benoit Laurent

**Affiliations:** ^1^ Department of Biochemistry and Functional Genomics, Faculty of Medicine and Health Sciences, Université de Sherbrooke, Sherbrooke, QC, Canada; ^2^ Medical Genetics division, Centre Hospitalier Universitaire de Sherbrooke (CHUS), Sherbrooke, QC, Canada; ^3^ Research Center on Aging, Centre Intégré Universitaire de Santé et Services Sociaux de l’Estrie-Centre Hospitalier Universitaire de Sherbrooke, Sherbrooke, QC, Canada

**Keywords:** reprogramming, urine, Fragile X syndrome, Sendai viral vectors, induced pluripotent stem cells

## Abstract

Human induced pluripotent stem cells (iPSCs) are a valuable tool for studying human development and diseases. iPSCs can be generated by reprogramming from any somatic cells, however establishing primary cell cultures can involve invasive procedures (e.g., skin biopsy) and be labor-intensive. In this paper, we describe an efficient, reliable, and non-invasive method for cultivating primary urine-derived cells (UDCs) and efficiently reprogram them into iPSCs using a feeder-free and non-integrative system. This approach has several advantages: (i) UDCs collection and culture are non-invasive, straightforward, and do not require medical personnel; (ii) reprogramming UDCs using commercially available Sendai viruses is highly efficient and reliable; and (iii) iPSCs generated from UDCs demonstrate strong differentiation potential. To showcase the effectiveness of this method, we generated iPSC lines from UDCs of three control individuals and three patients with Fragile X syndrome.

## 1 Introduction

The development of human induced pluripotent stem cells (iPSCs) in 2006 by Shinya Yamanaka was a groundbreaking discovery that revolutionized developmental biology since these cells emerged as a new model system offering unique opportunities to understand human embryonic development (Takahashi and Yamanaka, 2006; Takahashi et al., 2007). Somatic cells can be reprogrammed into iPSCs by ectopic expression of a core set of transcription factors consisting of OCT4, SOX2, KLF4, and c-MYC (OSKM). Since the initial reprogramming of human skin fibroblasts, hiPSCs have been generated from cells from multiple tissue and fluid types such as extraembryonic tissues, blood and urine (Huang et al., 2020; Tian et al., 2022; Gao et al., 2023). The reprogramming efficiency depends on the delivery mode of OSKM factors (episomal vectors, Sendai virus, adenovirus, etc.) and the somatic cell type (Rao and Malik, 2012; Cerneckis et al., 2024). The success rate varies around 0.01%–1% (Al Abbar et al., 2020).

In the field of neuroscience, iPSCs are widely used as tools to study early human neurodevelopment “in a dish” and model human pathologies such as neurodevelopmental disorders (NDDs). NDDs are a group of conditions characterized by atypical brain development that affects behavioral, cognitive and emotional functions (Morris-Rosendahl and Crocq, 2020). Among them, Fragile X syndrome (FXS) is the most prevalent cause of intellectual disability (ID) affecting 1 in 2,500–4,000 males and 1 in 7,000–8,000 females (Hagerman et al., 2017). FXS is caused by the epigenetic silencing of the Fragile X messenger ribonucleoprotein 1 (FMR1) gene encoding the FMRP protein. The absence of FMRP expression is thought to result in the characteristic FXS phenotypes. Individuals affected by FXS present moderate to severe ID along with other clinical manifestations such as autism spectrum disorder, anxiety and hyperactivity (Ciaccio et al., 2017). Current animal models are unable to fully replicate the human pathophysiology of FXS (Dahlhaus, 2018). Therefore, iPSCs derived from FXS individuals represent a powerful tool to study this NDD within an environment that recreates, with a high degree of fidelity, the complexity of human brain development and physiology (Lee et al., 2022).

Several studies have generated iPSCs by reprogramming fibroblasts or peripheral blood mononuclear cells (PBMCs) from FXS patients (Urbach et al., 2010; Doers et al., 2014; Bhattacharyya and Zhao, 2016; Achuta et al., 2017; Kurosaki et al., 2021). However, the combination of cognitive and behavioral impairments exhibited by FXS patients can make medical procedure such as skin biopsy or blood draws challenging to complete. Hence, collecting urine samples appears to be a valuable and non-invasive option to obtain somatic cells from FXS patients (Zafarullah et al., 2020).

The kidney contains tubules that reabsorb and return substances (e.g., water, electrolytes, nutrients) to the blood, but also eliminate the excess and wastes in the urine. Thousands of cells from this tubular system and downstream parts of the urinary tract (e.g., bladder, urethra) detach daily as part of the normal physiology and are excreted in urine (Lang et al., 2013). These urine-derived cells (UDCs) are fully viable and can be isolated from urine samples to be expanded in culture (Guan et al., 2014). In the context of FXS, UDCs can be collected anywhere without medical assistance and are a valuable cell source for cell reprogramming.

**FIGURE 1:**
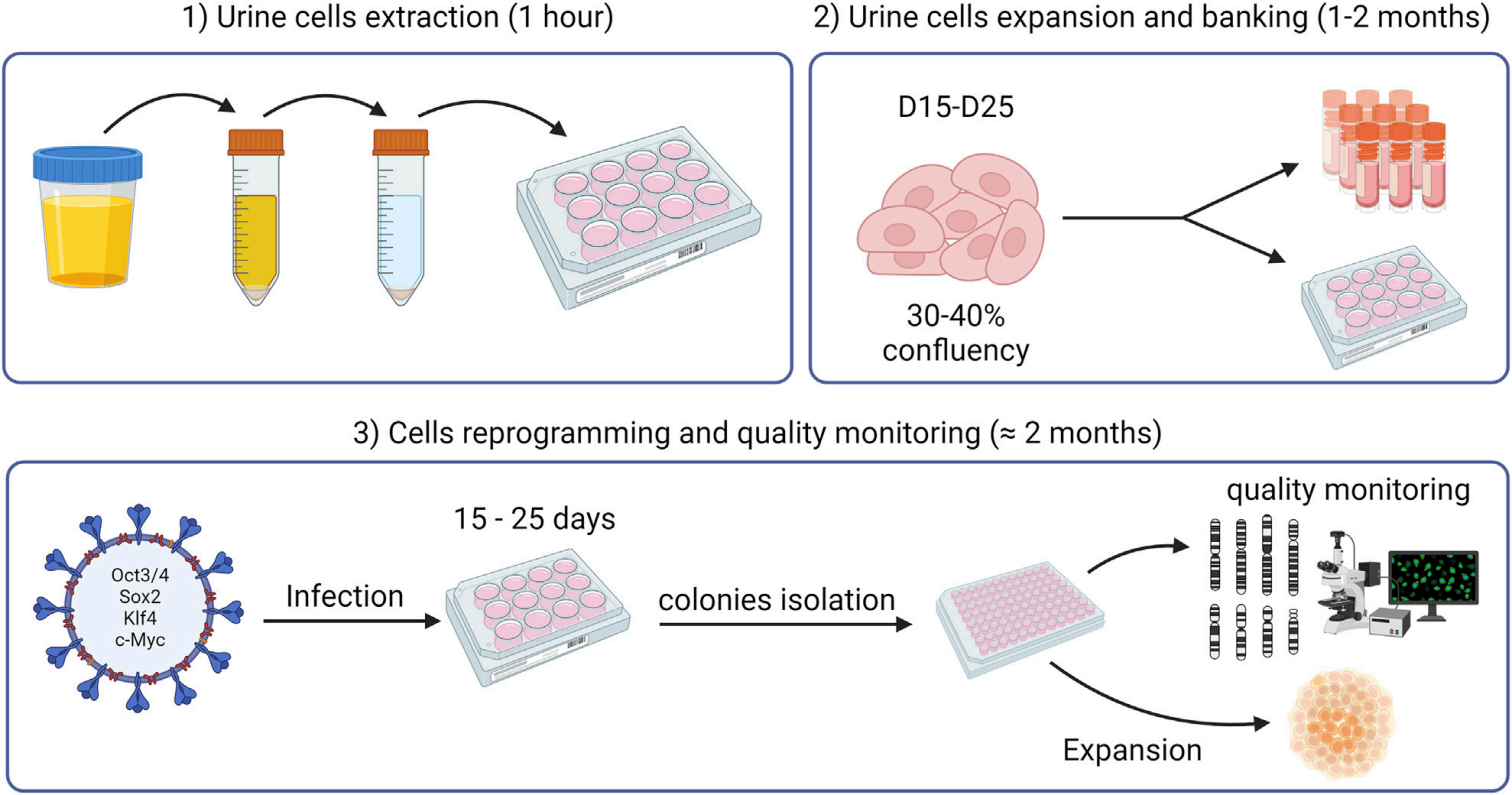
Schematic workflow of the methodology outlined in this paper.

We present here a protocol for efficient generation of hiPSCs from UDCs. To demonstrate the effectiveness of this method, we generated iPSCs from both control individuals and FXS patients. Non-integrating OSKM-coding viruses (i.e., Sendai viruses) were preferred for the cellular reprogramming as they circumvent risks of altered endogenous gene expression (Karami et al., 2023). A schematic workflow of the methodology described in this paper is depicted in Figure 1.

## 2 Materials and equipment

### 2.1 Reagents

*Caution.* Reagents for cell culture should be handled in an aseptic manner under laminar flow hood to prevent contamination.

#### 2.1.1 Culture of urine-derived cells

- REGM SingleQuot kit (Lonza #CC-4127)
- Renal epithelial cell basal medium (REBM) (Lonza #CC-3191)
- DMEM/F12 (Wisent #319-090-CL)
- Fetal bovine serum (FBS) (Wisent #080-150), aliquoted and stored at −20°C
- Penicillin-Streptomycin solution (Wisent #450-201-EL), aliquoted and stored at −20°C
- Amphotericin B (Wisent #450-105-QL), aliquoted and stored at −20°C
- Dulbecco’s Phosphate Buffered Saline (D-PBS) 1X, without calcium and magnesium (Wisent # 311-425-CL)
- 0.1% gelatine in sterile water (StemCell Technologies # 07903)
- Accutase (StemCell Technologies #07922), aliquoted and stored at −20°C

#### 2.1.2 hiPSC reprogramming and culture

- CytoTune-IPS 2.0 reprogramming kit (ThermoFisher Scientific # A16518)
- ReproTesR (StemCell Technologies # 05926)
- mTeSR1 (StemCell Technologies # 85850)
- Y-27632 dihydrochloride - Rho-Kinase inhibitor (Cayman Chemicals #1254)
- Accutase (StemCell Technologies #07922), aliquoted and stored at −20°C
- hESC-qualified Matrigel (Corning #354277)
- Cryostor CS10 (Stemcell Technologies #100-1,061)
- mFreSR (StemCell Technologies #05855)
- Trypan blue solution 0.4% (Gibco #15250061)
- Dulbecco’s Phosphate Buffered Saline (D-PBS) 1X, without calcium and magnesium (Wisent # 311-425-CL)

#### 2.1.3 Immunofluorescence

- Paraformaldehyde solution (16%) (ThermoFisher Scientific #28906)
- DAPI solution (1 mg/mL) (ThermoFisher Scientific #62248)
- Normal goat serum (NGS) (Wisent #053-210), aliquoted and stored at −20°C
- SlowFade Diamond Antifade Mountant (ThermoFisher Scientific #S36972)
- Clear nail polish
- 4-well chamber cell culture slides (ThermoFisher Scientific # 154526)
- Glass coverslips, 24 × 60 mm (ThermoFisher Scientific #12545M)
- Triton X-100 (Millipore Sigma #T8787)
- Dulbecco’s Phosphate Buffered Saline (D-PBS) 1X, without calcium and magnesium (Wisent # 311-425-CL)

#### 2.1.4 Functional assessment of hiPSC pluripotency

- STEMdiff Trilineage Differentiation Kit (Stemcell Technologies #05230)
- Trypan blue solution 0.4% (Gibco #15250061)
- Dulbecco’s Phosphate Buffered Saline (D-PBS) 1X, without calcium and magnesium (Wisent # 311-425-CL)
- Y-27632 dihydrochloride - Rho-Kinase Inhibitor (Cayman Chemicals #1254)
- Accutase (StemCell Technologies #07922), aliquoted and stored at −20°C
- hESC-qualified Matrigel (Corning #354277)
- mTeSR1 (StemCell Technologies # 85850)

#### 2.1.5 Assessment of sterility and *mycoplasma* testing

- SteriSEQ™ Rapid Sterility Testing Kit (ThermoFisher Scientific # A57186)
- MycoAlert^®^ *Mycoplasma* Detection Kit (Lonza # LT07-318)

### 2.2 Equipment

- 6-well tissue culture-treated plates (Corning #351146)
- 12-well tissue culture-treated plates (Corning #353043)
- 24-well tissue culture-treated plates (Corning #351147)
- Laminar flow hood (ThermoFisher Scientific #51029703)
- Adjustable micropipettes: P-2 (Gilson #FA10001M), P-20 (Gilson #FA10003M), P-200 (Gilson #FA10005M), and P-1000 (Gilson #F123602M)
- Filtered micropipette tips: 0.5-10 μL (FroggaBio #FT10), 2-20 μL (FroggaBio #FT20), 20-200 μL (FroggaBio #FT200), 100-1,000 μL (FroggaBio #FT1000)
- Serological pipettes, sterile: 2 mL (Sarstedt # 86.1252.001), 5 mL (Sarstedt # 86.1253.001), 10 mL (Sarstedt # 86.1254.001), 25 mL (Sarstedt # 86.1685.001)
- Conical tubes, sterile: 1.5 mL (Corning, cat. no. 3621), 15 mL (Corning #352096), 50 mL (Corning #352070)
- Cell culture incubator, CO2 at 5%, humidified at 37°C (ThermoFisher Scientific #51033546)
- Centrifuge (ThermoFisher Scientific #75009506)
- Water bath (37°C) (ThermoFisher Scientific #TSGP05)
- Hemacytometer (Millipore Sigma #Z359629-1EA)
- Urine collection container, 500 mL (Sarstedt # 75.9922.813)
- Cryogenic vials (Sarstedt # 72.380)
- Cell freezing container (ThermoFisher Scientific #5100-0001)
- 23G hypodermic needles (BD #305194)
- Syringe filters, Polyethersulfone (PES), pore size 0.22 μm (VWR #76479-016)
- Syringe, 20 mL sterile (BD #309661)
- Epifluorescence microscope (Zeiss Axioscope 2)

## 3 Preparation of reagents

### 3.1 Urine cell primary medium (UCPM)

This medium is composed of 500 mL of DMEM/F12 supplemented with 10% FBS, 1% penicillin/streptomycin, 1% amphotericin B and all supplements provided within the REGM SingleQuot kit. The medium is warmed at 37°C before use. All reagents are thawed overnight at 4°C. UCPM is stored at 4°C for up to two (2) weeks. For long term storage, UCPM is aliquoted in 50 mL canonical tubes and stored at −20°C.

### 3.2 Renal epithelial cell proliferation (REprolif) medium

This medium is composed of 500 mL of REBM supplemented with all supplements provided with the SingleQuot kit. The medium is warmed at 37°C before use. All reagents are thawed overnight at 4°C. The REprolif medium is stored at 4°C for up to two (2) weeks. For long term storage, aliquots in 50 mL canonical tubes are stored at −20°C.

### 3.3 ReproTesR medium

The supplement bottle is thawed at 4°C overnight and added to the basal media. The medium is warmed at 37°C before use. The ReproTesR medium is stored at 4°C for up to two (2) weeks. For long term storage, aliquots in 50 mL canonical tubes are stored at −20°C.

### 3.4 mTesR1 medium

The supplement bottle is thawed at 4°C overnight and added to the basal media. The medium is warmed at 37°C before use. The mTesR1 medium is stored at 4°C for up to two (2) weeks. For long term storage, aliquots in 50 mL canonical tubes are stored at −20°C.

### 3.5 Y-27632 stock solution

The Y-27632 Rho-Kinase inhibitor is dissolved in D-PBS to a stock concentration of 10 mM (1,000X) and sterilized using a 0.22 μm syringe filter. Aliquots in 1.5 mL microtubes are stored at −20°C.

### 3.6 Plate coating with gelatine

A 0.1% gelatine solution is dispended into a culture plate for well coating, with usually 0.5 mL per well for a 12-well plate and 1 mL per well for a 6-well plate. Plates are incubated at 37°C for a minimum of 30 min and then washed twice with D-PBS before use.

### 3.7 Plate coating with matrigel

The Matrigel bottle is thawed overnight on ice in a refrigerator. The dilution factor, specified on the lot-specific Certificate of Analysis, is calculated for each lot of Matrigel, based on the protein concentration. Aliquot volume is typically between 270-350 µL and aliquots of the Matrigel solution are stored in 1.5 mL microtubes at −20°C. One aliquot of Matrigel is added to 25 mL of DMEM/F-12 medium to coat 6-well plates (1 mL/well) or 12-well plates (0.5 mL/well). The cultureware is incubated at room temperature (15°C-25°C) for at least 1 h before use. The liquid remaining in each well is aspirated immediately before use. It is important to ensure that the Matrigel solution covers the entirety of the well surface and that the tip of the pipette does not scratch the coated surface.

*Critical point.* It is critical to always keep the Matrigel on ice as it will solidify beyond 10°C. Matrigel-coated plates are stored at 4°C for up to two (2) weeks. Refrigerated plates are wrapped in parafilm to prevent Matrigel solution evaporation.

### 3.8 Normal goat serum solution

To perform the immunofluorescence, the NGS is diluted to a final 10% concentration with D-PBS. The diluted solution is kept at 4°C for up to one (1) week.

### 3.9 STEMdiff trilineage mediums

The differentiation medium bottles (ectoderm, mesoderm, endoderm) are thawed overnight at 4°C. Mesoderm and endoderm mediums is stored at 4°C for up to two (2) weeks, while ectoderm medium is stored at 4°C for up to one (1) week. Mediums is aliquoted in 50 mL conical tubes and stored at −20°C.

## 4 Methods

The methods related to UDC extraction, culture and biobanking as well as cell reprogramming, and quality monitoring (Figure 1) are described in detail below.

### 4.1 UDC extraction (TIMING ∼1 h)

1. Urine samples are collected into a sterile container and kept at room temperature (15°C-25°C) prior to their processing. To increase the volume of urine to be collected, participants are advised to drink a full bottle of water (500 mL) 1-2 h before collection. Participants are also instructed to clean their ureteral aera with a disinfectant wipe just before collection.
2. Urine sample is split into 50 mL conical tubes under the cell culture hood and then centrifuge at 400 g for 5 min at room temperature (15°C-25°C).
3. Supernatants are removed by aspiration to leave approximately 1 mL of urine above each cell pellet. UDCs are resuspended by pipetting up and down and transferred into the same 50 mL conical tube.
4. Each conical tube is washed with 5 mL of D-PBS that are then transferred to the tube containing the pooled cell suspensions. D-PBS is added to this collection tube to a final volume of approximately 45 mL.
5. After a centrifugation at 400 g for 5 min at room temperature (15°C-25°C), the supernatant is removed, and cells are resuspended in 2 mL of UCPM. Cells are put into a gelatin-coated plate and incubated at 37°C for 24 h. The culture is now at the passage 0 (P0).

*Critical point*. It is important to put liquid against the well wall, and not directly on the well, to avoid any disruption of the gelatine coating. The use of a unique well from a 12-well plate per participant is recommended for promoting UDC expansion.

### 4.2 UDC expansion and banking (TIMING ∼ 1-2 months)

#### 4.2.1 UDC culture

*Critical point*. UDCs take several days to become adherent to the culture plate. It is hence crucial to handle plates both minimally and gently. It is also equally vital not to remove any medium until day 4.

1. From day 1 to day 3, 1 mL of UCPM is gently added each day to the UDC culture.
2. At day 4, the majority of culture medium (approximately 4 mL) is removed with a serological pipette, to leave around 1 mL of medium in the well. Then, 1 mL of REproliff medium is carefully added to the well.
3. At day 5 and beyond, half of the medium is changed with REproliff every day. It is recommended to observe the UDC culture regularly under the microscope to monitor the apparition of colonies. UDC colonies should be visible within the initial two (2) weeks of the culture. The number of colonies per culture is highly variable, usually ranging from four (4) to ten (10). UDCs are ready for passage when they cover roughly 30%-40% of the well surface, usually occurring within 10–20 days after the extraction.
4. When UDCs at P0 are ready to passage (30%-40% confluency), the culture medium is removed, and cells are washed once with D-PBS. 0.5 mL of accutase is added on cells which are then incubated at 37°C for 5 min 1.5 mL of D-PBS is next added to dilute the accutase and the cell suspension is transferred to a 15 mL conical tube before a centrifugation at 400 g for 5 min at room temperature (15°C-25°C). The well should be washed thoroughly to harvest as much cells as possible. Cells are resuspended in 1 mL of warm REproliff medium and seeded in one well of a 12-well gelatine-coated plate (1:1 split ratio). The cultureware is incubated at 37°C and cells are now at passage 1 (P1).
5. Change medium with warm REproliff every other day until UDCs reach a confluency of 70%-90%. For the passage 2 and beyond, use the protocol described above but with a 1:4 split ratio.

*Critical point.* The accutase solution is warmed to 37°C before use. It is important to prepare a gelatine-coated plate before passaging UDCs.

#### 4.2.2 UDC cryopreservation

1. The culture medium is removed, and cells are washed once with D-PBS. 0.5 mL of accutase is added on cells which are then incubated at 37°C for 5 min.
2. 1.5 mL of D-PBS is next added to dilute the accutase and the cell suspension is transferred to a 15 mL conical tube before a centrifugation at 400 g for 5 min at room temperature (15°C-25°C).
3. The supernatant is discarded and UDCs are resuspended in cold (4°C) Cryostor CS10 cell freezing medium before to be transferred to cryogenic vials. We recommend using one (1) cryogenic vial per well.
4. Vials are put into the cell freezing container and incubated at −80°C overnight before to be transferred to liquid nitrogen for optimal long-term storage.

*Critical point.* The accutase solution is warmed to 37°C before use. Cells are cryopreserved upon reaching confluency at P1 or after subsequent passages.

#### 4.2.3 UDC thawing

1. A cryogenic vial containing UDCs is thawed in a water bath at 37°C until only small ice crystals remain, and cells are then transferred in a 15 mL conical tube containing 3 mL of warm REproliff medium.
2. After a centrifugation at 400 g for 5 min at room temperature (15°C-25°C), UDCs are resuspended in warm REproliff medium and seeded on a gelatine-coated plate. If cells were cryopreserved at the density described above, a 1:2 split ratio can be used. The cultureware should be carefully homogenized to disperse cells before incubation at 37°C.
3. Medium change is then performed every other day with REproliff medium until UDCs reach a 70%-90% confluency.

### 4.3 UDC reprogramming (TIMING ∼1 month)

*Critical point*. A successful hiPSC generation highly depends on having a high-quality UDC culture characterized by healthy cell morphology and robust proliferation. We advise initiating the UDC reprogramming process prior to reaching passage 5 (P5), as UDCs tend to enter a senescent state beyond this point. UDCs can be reprogrammed following cryopreservation. We did not observe any significant alterations in reprogramming efficiency following a cryopreservation. *Mycoplasma* contamination can occur in UDC culture and should be assessed before initiating reprogramming to prevent subsequent problems. Please refer to the section 4.5.4 for the experimental procedure.

1. UDCs are seeded at 25,000–50,000 cells per well on a gelatine-coated 6-well plates usually two (2) days before infection (D-2). We recommend preparing at least three (3) wells of a 6-well plate to guarantee an optimal reprogramming. One of these wells will be used to estimate the number of cells to be infected.
2. On the day of infection (D0), cells should reach a 30%-50% confluency. One of the wells is used to estimate the number of cells to be infected using any cell counting method (e.g., Trypan Blue exclusion). The volume of each viral vector is calculated with this formula:

$$\text{Volume of viral vector }\left(\mu l\right)=\left(\frac{\text{MOI}\;x\;\text{number}\;\text{of}\;\text{cells}}{\text{viral}\;\text{vector}\;\text{titter}}\right)\;x10^{-3}$$

The titer of each Sendai reprogramming vector is lot-dependent. The Certificate of Analysis indicates the titer of the three Sendai reprogramming vectors: hKOS, hC-MYC, hKLF4. Their respective multiplicity of infection (MOI) should be 5-5-3 (i.e., hKOS MOI = 5; hC-MYC MOI = 5; hKLF4 MOI = 3).

3. On the day of infection (D0), UDCs are transduced with the Sendai reprogramming vectors at the appropriate MOI. To do so, each viral vector is thawed by immersing in a 37°C water bath for 5 s and then placed on ice until fully thawed. After thawing, the pre-calculated volume of each viral vector is added to the REprolif medium. Use 1 mL of medium per well to be infected. The virus-containing medium is added on UDCs, which are then incubated at 37°C.

*Critical point.* All procedures should be performed rapidly once viral vectors are thawed. Viral vectors should be aliquoted in microtubes and store at −80°C once thawed for the first time. The volume of aliquots depends on the titter of each Sendai reprogramming vector. Viral vectors aliquots should not be thawed more than once.

4. At day 1 (D1), 24 h after infection, the culture medium is replaced with fresh warm REprolif medium. The medium is changed every day with REprolif afterwards from D2 to D6. Infected UDCs are cultured on the same plate until D7, even if they reach full confluence.
5. At day 7 (D7), infected cells are passaged on a Matrigel-coated plate using accutase as previously described. Cells are seeded at 50,000 to 100,000 per well of a 6-well Matrigel-coated plate in REprolif medium.
6. At day 8 (D8), the REprolif medium is changed for the ReproTesR medium. The ReproTesR medium is changed every other day. Cultures should be observed regularly under the microscope to monitor the emergence of hiPSC colonies.

**FIGURE 2:**
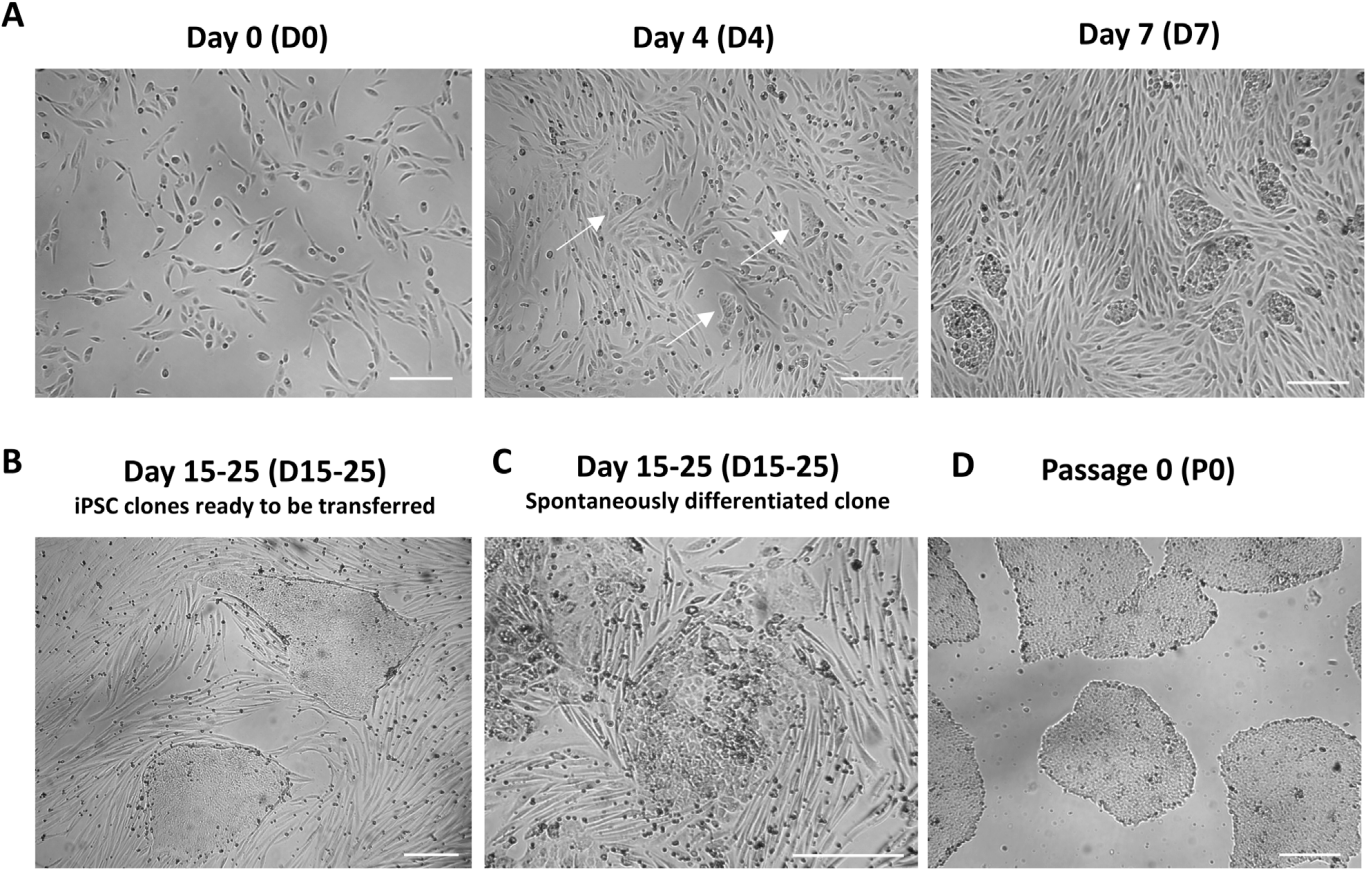
UDC reprogramming **(A)** UDCs at day 0 (D0) following infection with Sendai viruses. Urine cells should be at a confluency of 30%-50% after plating, prior to infection. UDCs undergoing reprogramming, characterized by small clumps of densely packed cells, should be visible day 4 (D4) after plating. The clumps of UDCs undergoing reprogramming are larger and more abundant at day 7 (D7) following infection. **(B)** Representative image of fully reprogrammed iPSC colonies (usually 15–25 days after transfer on Matrigel-coated plates). These colonies exhibit a typical iPSC colony morphology with a smooth border and high cell density. **(C)** Representative image of iPSC colonies experiencing spontaneous differentiation. **(D)** Example of a healthy iPSC culture following subclone isolation of Matrigel. Scale bar = 250 µm.

*Critical point*. UDC reprogramming is characterized by cell clumps that should emerge few days following plating on Matrigel (Figure 2A). iPSC colonies are ready to transfer when they reach a diameter of around 500-750 μm and display typical healthy morphology i.e., smooth border, high cell density, high nucleus/cytoplasm ratio (Figure 2B). These colonies are typically observed 7–14 days following plating on Matrigel. It is normal to observe some colonies that will undergo spontaneous differentiation (Figure 2C) but it is important not to subculture these colonies.

7. Starting day 15 (D15) until day 25 (D25), hiPSC colonies can be isolated. Using a 23G needle, a grid pattern of 6 squares is made on a selected iPSC colony to ensure that a fragment of the appropriate size will be transferred. Each piece of the iPSC colony is picked with a P200 micropipette and transferred to a well of a 12-well Matrigel-coated plate containing 1 mL of mTeSR1 medium supplemented with 10 μM Y-27632. Using a P1000 micropipette, up and down cycles are performed to ensure breakage of big hiPSC aggregates. The iPSC clone is then incubated at 37°C and is now at passage 0 (P0) (Figure 2D).

### 4.4 Clone expansion and banking (TIMING ∼1 month)

1. The iPSC clone at P0 is cultured in mTeSR1 medium (with a daily medium change) until colonies reach a diameter of around 1–2 mm. Cells are then passaged with accutase as previously described and cultured on 6-well Matrigel-coated plates in mTeSR1 medium supplemented with 10 μM Y-27632. Cells from one well of a 12-well plate are usually seeded in one well of a 6-well plate.
2. To expand hiPSC clones, cells should reach a 70%-80% confluency. Cells are then seeded on Matrigel-coated plates using mTeSR1 medium supplemented with 10 μM Y-27632 at a 1:6 to 1:10 split ratio.

*Critical point*. hiPSC clones can be cryopreserved once they reach confluency at P1. It is important to start cryopreservation as soon as possible in order to ensure banking of iPSC clones at low passages.

3. To cryopreserve hiPSC clones, the mTeSR1 medium is removed and cells are washed with D-PBS. 1 mL of accutase per well is added on cells which are then incubated at 37°C for 5 min 1.5 mL of D-PBS is next added to dilute the accutase and the cell suspension is transferred to a 15 mL conical tube before a centrifugation at 400 g for 5 min at room temperature (15°C-25°C). Cell pellet is resuspended in 1 mL of cold mFreSR medium and transferred into a cryovial. We recommend using two (2) cryovials per well. Cryovials are put into a cell freezing container and then incubated at −80°C overnight. The next day, cryovials are transferred into liquid nitrogen for optimal long-term storage.

### 4.5 Validation of iPSC clones (TIMING ∼1 week)

#### 4.5.1 Assessment of pluripotent stem cells marker expression by immunofluorescence

*Critical point*. hiPSCs must be cultured on Matrigel-coated cell culture chamber slides or coverslips in order to perform immunofluorescence staining and analyses.

1. Fixation: Cells are fixed by removing the culture medium and adding to each chamber warm mTesR1 medium supplemented with 4% PFA. Cells are incubated at 37°C for 20 min and are then washed three (3) times with D-PBS, incubating each wash for 5 min.
2. Blocking and permeabilization: After the D-PBS is removed, a blocking solution composed of 10% NGS and 0.1% Triton X-100 is added in each chamber. Slides or coverslips are incubated at room temperature (15°C-25°C) for a minimum of 30 min.
3. Primary antibody hybridization: A primary antibody solution is prepared by diluting the primary antibody in 10% NGS. The expression of at least two (2) of the following iPSC markers should be assayed: OCT4, SOX2, Nanog, KLF4, TRA-1-60, TRA-1-81, SSEA1, SSEA4 (Adewumi et al., 2007; Andrews and Gokhale, 2024). The reference and dilution of each primary antibody used in this study are listed in Table 1. The blocking solution is then removed and a sufficient volume of primary antibody solution (usually 75 μL) is added to cover the surface of each chamber. Slides or coverslips are incubated at 4°C overnight.
4. Secondary antibody hybridization: The primary antibody solution is removed from each chamber and three (3) washes with D-PBS are performed by incubating each wash for 5 min at room temperature (15°C-25°C). The secondary antibody solution is prepared by diluting each fluorochrome-conjugated secondary antibody in 10% NGS. The reference and dilution of each secondary antibody used in this study are listed in Table 1. The D-PBS is then removed and a sufficient volume of secondary antibody solution (usually 75 μL) is added to cover the surface of each chamber. Slides or coverslips are incubated at room temperature (15°C-25°C) for 60 min.

**TABLE 1:** Antibodies used for the assessment of hiPSC pluripotency and functionality.

Protein	Reference	Application	Dilution
Sox2	Santa Cruz Biotechnology (#sc-365823)	iPSC marker	1/100
Oct4	Cell Signaling Technology (#2840)	iPSC marker	1/250
Nanog	Abclonal (#A3232)	iPSC marker	1/500
FMRP	Cell Signaling Technology (#4317)	FXS marker	1/500
Pax6	Biolegend (#901301)	Ectoderm marker	1/500
Nestin	Santa Cruz Biotechnology (#sc23927)	Ectoderm marker	1/100
FOXA2	Cell Signaling Technology (#8186)	Endoderm marker	1/400
SOX17	Biolegend (#698501)	Endoderm marker	1/500
NCAM	Stem Cell Technologies (#60021)	Mesoderm marker	1/500
Brachyury	Abclonal (#A16887)	Mesoderm marker	1/200
Anti-Rabbit IgG 488nm	Cell Signaling Technology (#4412)	Secondary antibody	1/1,000
Anti-mouse IgG 647nm	Cell Signaling Technology (#4410)	Secondary antibody	1/1,000

*Critical point*. Cells must be protected from light from step 4 and onwards to avoid fluorochrome bleaching..

5. DAPI counterstaining: The secondary antibody solution is removed from each chamber and three (3) washes with D-PBS are performed, incubating each wash for 5 min at room temperature (15°C-25°C). A sufficient volume of DAPI solution (1 μg/mL) is prepared by diluting the stock solution (1 mg/mL) in D-PBS. After D-PBS removal, a sufficient volume of DAPI solution is added to cover the surface of each chamber. Slides or coverslips are incubated at room temperature (15°C-25°C) for 10 min.
6. Slide mounting: The DAPI solution is removed from each chamber and two (2) washes with D-PBS are performed, incubating each wash for 5 min at room temperature (15°C-25°C). The third wash is next performed with deionized water. The culture chamber from each slide is detached following the manufacturer’s instructions. A sufficient volume of SlowFade liquid mounting medium is applied to cover the surface of the slide. A coverslip is gently positioned onto the slide and nail polish is applied along the slide’s perimeter to secure it. After drying for 60 min at room temperature (15°C-25°C), slides can be stored at 4°C.
7. Slides are finally observed with an epifluorescence microscope or other fluorescence microscope system to acquire images from different randomly selected fields using a ×10 objective.

#### 4.5.2 Assessment of genomic stability by G-banded karyotyping

*Critical point*. The reliability of G-banded karyotyping is significantly influenced by the sample preparation protocol. As the karyotype preparation and analysis is complex, karyotyping should be conducted by trained specialists within a certified laboratory to ensure result reliability. All G-banded karyotypes performed on the iPSC lines described in this study were performed by the clinical cytogenetics laboratory of the Sherbrooke University hospital center in accordance with the standards laid down by the Canadian College of Medical Geneticists. This laboratory has an ISO15189 accreditation. The procedure is summarized below.

When reach 50% of confluency, iPSCs are treated with colcemid (50 ng/mL) for 1 hour before being washed with D-PBS and harvested with accustase. Collected cells are centrifuged at 400 g for 5 min at room temperature (15°C-25°C) and the cell pellet is then suspended in 1 mL of D-PBS. Subsequently, 12 mL of warm 0.4% KCl solution is added dropwise to the tube while carefully shaking it. Cells are next incubated at 37°C for 15 min. Cells are then centrifuged at 400 g for 5 min at room temperature (15°C-25°C) and resuspended in 1.5 mL of D-PBS. 12 mL of Carnoy I solution (25% glacial acetic acid, 75% methanol) was then added dropwise to fix the cells. The cell suspension was incubated at room temperature (15°C-25°C) for 10 min and then centrifuged at 400 g for 5 min at room temperature (15°C-25°C). The fixation procedure was repeated two (2) more times. Afterwards, the cells were resuspended in 1 mL of Carnoy I solution and stored at −20°C. Fixed cells were subsequently spread on a microscope slide and treated with 0.125% of trypsin diluted in 0.9% NaCl for 30 s. Slides were washed two (2) times with 0.9% NaCl before being stained with Gurrs Giesma stain solution (6% Gurrs Giesma stain, 2% acetone in Gurrs 6.8 buffer) for 5 min. Slides were then washed two (2) times with Gurrs 6.8 buffer. Slides can be coverlipped with mounting reagent, prior to microscope examination (optional). Metaphase images are automatically captured using the Metafer software (MetaSystems) and karyotyped with the Ikaros software (MetaSystems). Around 8 to 17 cells are analyzed with a resolution of 350-425 bands per haploid metaphase.

#### 4.5.3 Functional assessment of pluripotency

*Critical point.* iPSC culture should be at a confluency of 70%-80% and colonies must display healthy morphology before starting this procedure. Also make sure that Matrigel-coated cell culture chamber slides or coverslips are ready to use before starting this procedure..

1. Cell plating (D0): Cells are harvested using accutase i.e., 1 mL for a well of 6-well plate with a 5 min-incubation at 37°C. Two (2) volumes of D-PBS are then added to dilute the accutase and cell suspension is transferred to a 15 mL conical tube before a centrifugation at 400 g for 5 min at room temperature (15°C-25°C). Cells are then resuspended in 1 mL of mTesR1 supplemented with 10 µM Y-27632 and cells are counted using Trypan blue exclusion. Following cell densities in Table 2, the appropriate volume of cell suspension is added into a well of a Matrigel-coated culture chamber and completed up to 0.5 mL of mTesR1 supplemented with 10 µM Y-27632. Plates are shaken well to disperse the cells and then incubated at 37°C.
2. Lineage-specific differentiation: On day 1 (D1), the medium is removed and cells are replaced with lineage-specific medium. A daily medium change is performed with the appropriate medium until day 5 (D5) for mesoderm and endoderm differentiation. For ectoderm differentiation, the culture is extended until day 7 (D7).
3. Assessment of lineage differentiation: The differentiation efficiency of each germ lineage is assessed through the detection of lineage-specific markers. Immunofluorescence experiments are performed as described in Section 4.5.1 using antibodies directed against the markers listed in Table 3. The reference and dilution of each primary antibody used in this study are listed in Table 1.

**TABLE 2:** Seeding densities for each embryonic lineage differentiation.

Lineage	Cell density (cells/cm^2^)
Mesoderm	50,000
Ectoderm	200,000
Endoderm	200,000

**TABLE 3:** Markers used to validate the differentiation of embryonic lineages.

Lineage	Markers
Mesoderm	Brachyury, NCAM
Ectoderm	PAX6, Nestin
Endoderm	SOX17, FOXA2

#### 4.5.4 Assessment of sterility and *mycoplasma* testing

*Critical point.* Biological contaminations from bacteria, fungi, and *mycoplasma* are a significant treat to iPSC cultures and can affect their differentiation. Such contamination can significantly undermine the validity of the results obtained from infected iPSCs. Therefore, sterility and *mycoplasma* testing should be regularly conducted (especially in time of increase cell banking) to ensure the maintenance of cell line quality.

Biological contamination by bacteria and fungi was tested using the qPCR-based SteriSEQ™ Rapid Sterility Testing Kit and *mycoplasma* contamination using the luciferase-based MycoAlert^®^ *Mycoplasma* Detection Kit. All assays were conducted following the manufacturer’s protocol.

## 5 Anticipated results

This protocol was used to generate six iPSC lines from urine samples provided by three control individuals and three patients with FXS. These specific cell lines served as examples of the anticipated outcomes for this methodology, and all validation data are included in this paper. Demographic details of the donors are listed in Table 4. The recruitment was performed through the Fragile X Clinic, at the CIUSSS de l’Estrie-CHUS (Sherbrooke, Québec, Canada) following the protocol (protocol 2020–3616) approved by the Ethics Review Board of the CIUSSS de l’Estrie-CHUS. Informed written consent was obtained from healthy controls and from a caregiver for FXS donors.

**TABLE 4:** Demographic characteristics of the donors.

Cell line	Sex	Age	Group
IPS_C1	M	27	CTL
IPS_C2	F	22	CTL
IPS_C3	M	14	CTL
IPS_X1	M	12	FXS
IPS_X2	F	29	FXS
IPS_X3	F	23	FXS

### 5.1 UDC extraction and culture

The volume of urine collected is usually correlated to the successful culture of UDCs. Therefore, participants should provide as much urine as possible. Sampling of 200-500 mL of urine is typically sufficient to ensure a viable UDC culture. It is essential to process urine samples promptly after collection to favor cell survival and prevent decay due to extended processing times. We do recommend placing urine samples on ice, as it promotes the formation of aggregates within the specimen. Alternatively, multiple urine samples from the same donor can be collected during the same day to increase the likelihood of successfully establishing a UDC culture.

**FIGURE 3:**
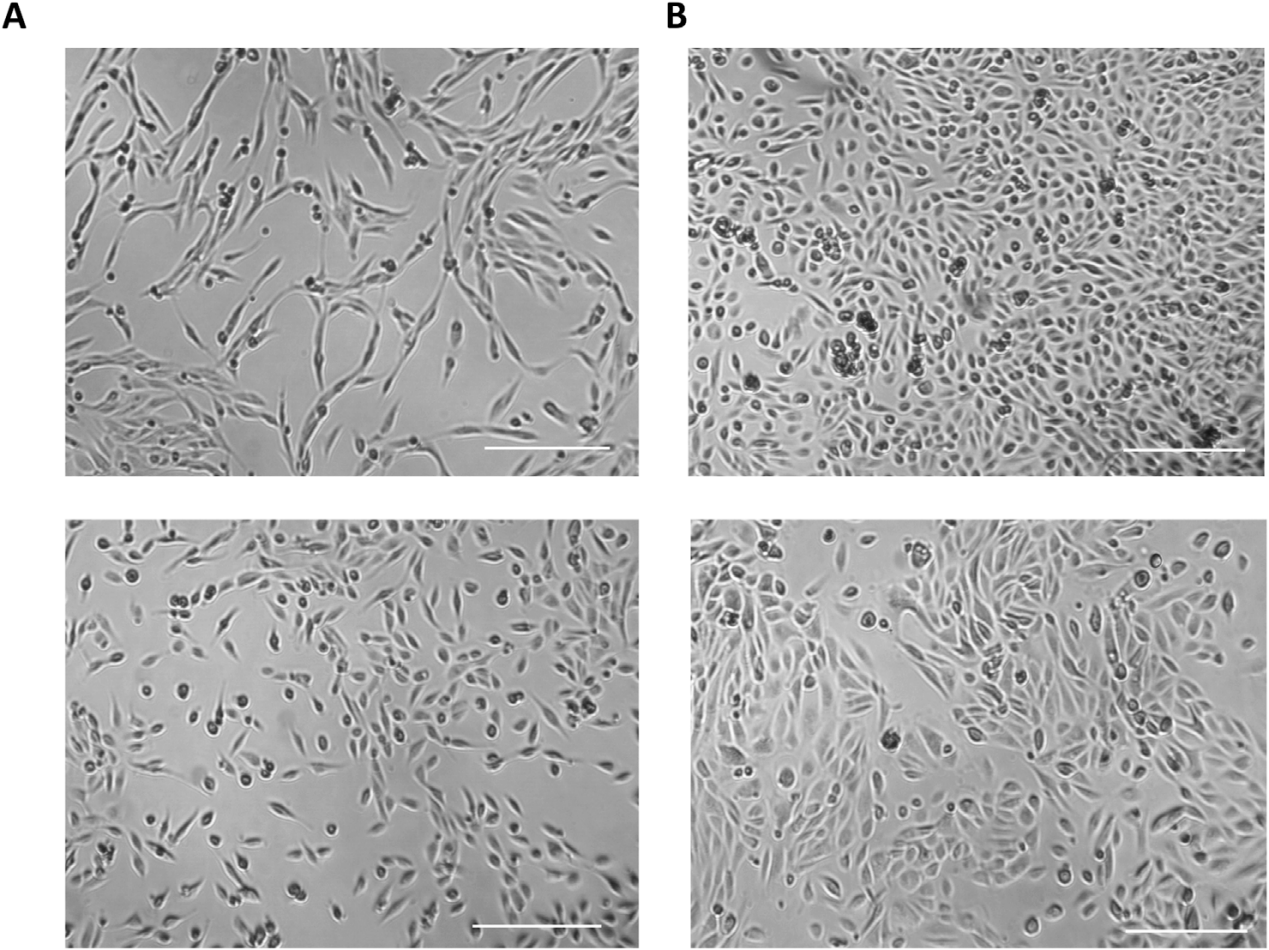
Types of UDCs extracted and cultured from urine samples. **(A)** Type I cells exhibit a spindle-like morphology and originates from the renal mesenchyme. **(B)** Type II cells have a cobblestone-like morphology likely originating from the nephron tubule. Scale bar = 250 µm.

Two distinct types of UDCs are extracted and cultured from urine samples and can be easily distinguished by their morphology: Type I exhibits a spindle-like morphology (Figure 3A, top panel), while Type II exhibits a cobblestone-like morphology (Figure 3A, bottom panel) (Dörrenhaus et al., 2000; Chen et al., 2020). Characterisation through marker expression analysis reveal that both types are composed or cells from the renal epithelium, with type I UDCs originating from the renal mesenchyme and type II from the nephron tubule (Dörrenhaus et al., 2000; Rahmoune et al., 2005; Zhou et al., 2011; Chen et al., 2020; Zafarullah et al., 2020).

UDCs obtained from freshly collected urine samples exhibit significant heterogeneity. For instance, cultures derived from female individuals and patients typically exhibit a high quantity of spherical cells, primarily consisting of squamous cells originating from the vagina and the urethra (Supplementary Figures 1SA, SB), while male samples exhibit a limited cell count (Supplementary Figure S1C) (Culenova et al., 2021). These cells do not adhere to the culture plate and will be gradually eliminated during medium changes. Some urine cultures may also contain non-biological objects (Supplementary Figure S1D). The presence of these elements does not usually appear to have a detrimental impact on the success rate of establishing UDC cultures.

The initial UDC colonies should become visible within the first 2 weeks of culture. The number of colonies can vary significantly between cultures, but typically falls within the range of 4–10 colonies per 250 mL of urine. Urine cells at passage 0 (P0) are considered ready for passaging when they cover approximately 30%-40% of the well surface, a milestone usually achieved within 10–20 days of culture. At this stage, it is common to observe a relatively dense population of cells within colonies. Urine cells at passage 1 (P1) and beyond exhibit robust proliferation, and they are ready for passaging when they reach a confluency of 70%-90% what usually occurs once or twice a week. The two distinct UDC types i.e., cells with a cobblestone-like morphology and with a spindle-like morphology (Figure 3), can be cultured for several passages, but spindle-like cells demonstrate a superior proliferative capability, allowing culture them up to passage 10 (P10). In contrast, cobblestone-like cells proliferate less and undergo senescence around passage 5 (P5) (Shi and Cheung, 2021).

### 5.2 UDC reprogramming and iPSC clone expansion

Successful UDC reprogramming highly depends on the quality of the cells to be reprogrammed. Therefore, we recommend initiating reprogramming before UDCs reach passage 5 (P5). Importantly, UDCs can be successfully reprogrammed after cryopreservation without impact on reprogramming efficiency.

On the day of infection, UDCs should ideally have a 30%–50% confluency. Following infection with Sendai viruses, cells undergoing reprogramming will appear within 3–4 days as small clumps of densely packed cells (Figure 2A). Their size should increase by the day 7 (D7) post-infection at which point they are transferred to Matrigel-coated plates to support the growth and the undifferentiated state of newly reprogrammed iPSC clones (Figure 2A). When a cluster colony acquires a morphology typical of iPSC colony (i.e., smooth border, high cell density, high nucleus/cytoplasm ratio) and reaches a 500-750 µm diameter (typically 15-25 days following infection), they are ready to be picked manually and transfer on Matrigel (Figure 2B). At least 20 to 30 clones should be isolated over the span of 3–5 days to enhance the subcloning success. A notable portion of iPSC subclones may fail to adhere to plates or undergo spontaneous differentiation after transfer (Figure 2C).

**FIGURE 4:**
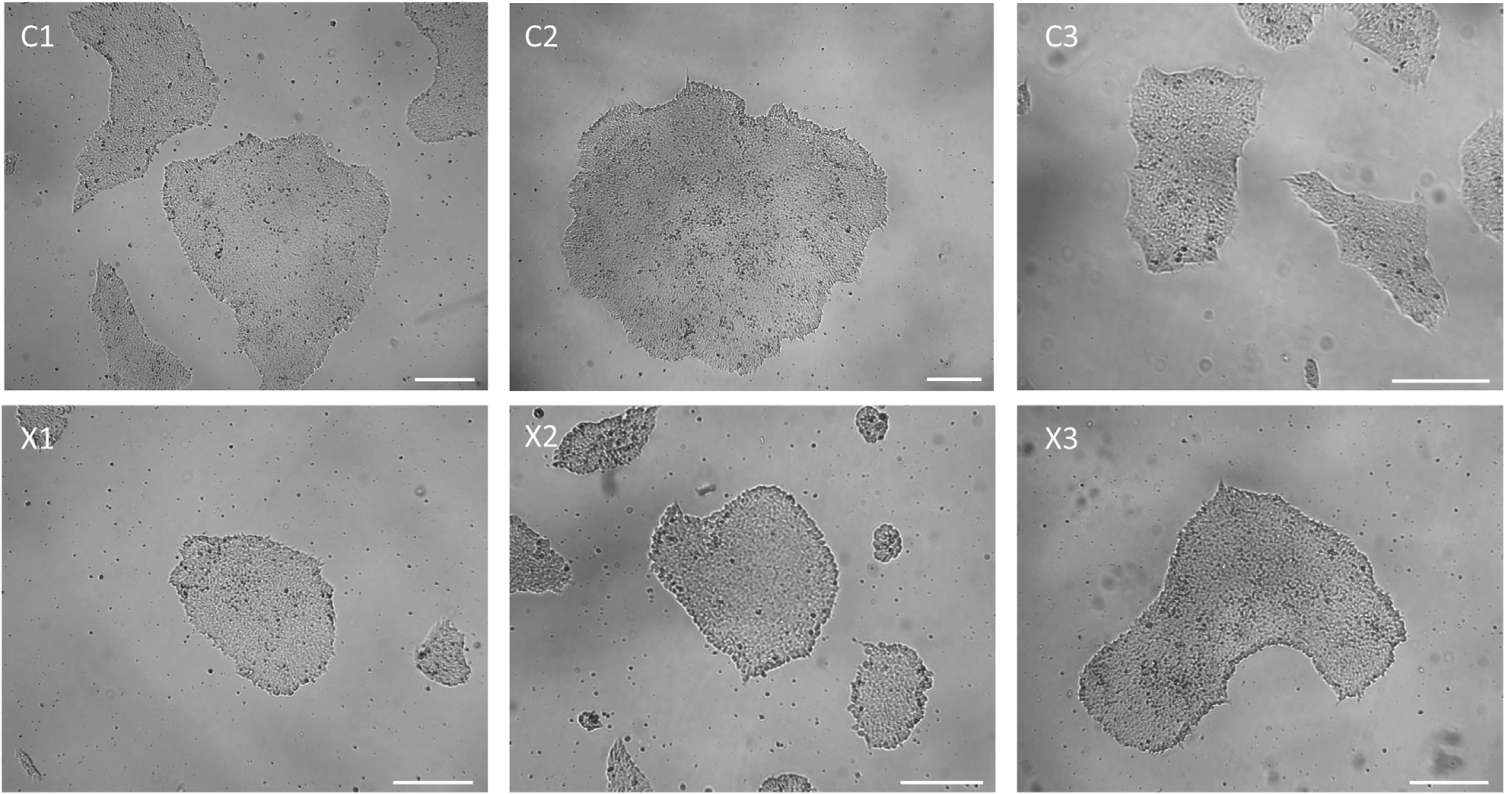
Representative colony morphology of all iPSC lines generated for this paper. Scale bar = 250 µm.

After manual isolation of several clones in independent wells (passage 0), newly reprogrammed iPSCs are let grow until they reach a 1-2 mm diameter. Starting from passage 1 (P1) and beyond, hiPSC clones should be passaged when they attain a 70%-80% confluency. A high-quality iPSC culture is defined by the presence of more than 95% of colonies exhibiting typical iPSC morphology. This morphology is characterized by a high cell density, a prominent nucleus to cytoplasm ratio, and smooth borders (Wakui et al., 2017) (Figures 2D, 4). During the expansion, some cells within the center the colonies can undergo spontaneous differentiation (Figure 2C). These spontaneously differentiated cells can be manually remove using a P200 pipettor or a 23G needle.

### 5.3 iPSC line validation

#### 5.3.1 Assessment of pluripotency stem cell marker expression

**FIGURE 5:**
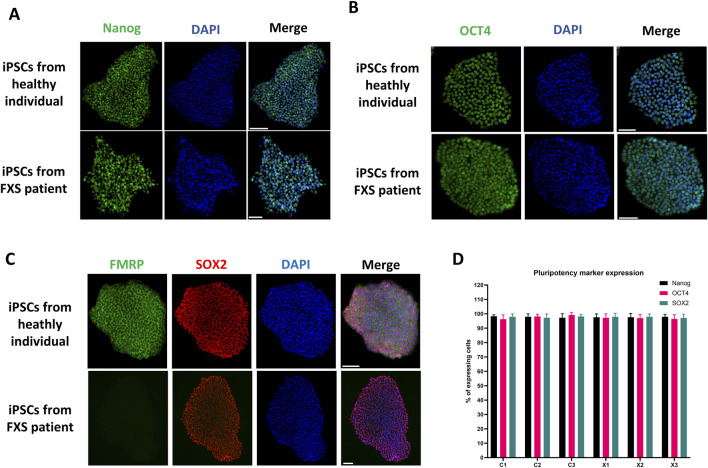
Assessment of pluripotency markers and FMRP expression in iPSC lines. Representative immunostaining of the pluripotent stem cell markers Nanog **(A)**, OCT4 **(B)**, and SOX2 **(C)** as well as FMRP **(C)** in a control and a FXS iPSC line. Immunostaining of all iPSC lines produced in this paper can be found in Supplementary Figure S2. Scale bar = 100 µm. **(D)** Quantification of pluripotency markers expression in all iPSCs lines (n = 10 fields of views per cell line, mean ± SD).

In a high-quality iPSC culture, it is expected that most cells (over 95%) express pluripotent stem cell markers such as Nanog, Oct4 and Sox2. If a smaller proportion of cells exhibit the expression of these markers, it could indicate a loss of pluripotency or incomplete reprogramming of the clone. However, the expression of these markers does not prove pluripotency but rather suggests that the iPSC culture remains in an undifferentiated state. Figure 5 shows a representative example of immunostaining for Nanog, Oct4 and Sox 2 pluripotency markers as well as FMRP in iPSC lines derived from healthy individuals and FXS patients. The results from all cell lines in this study demonstrated pluripotency marker expression in over 95% of cells (Figure 5D). Control iPSCs showed FMRP expression, while FXS iPSCs did not. Detailed data for all cell iPSC lines are presented in Supplementary Figure S2.

#### 5.3.2 Assessment of genomic integrity and stability

**FIGURES 6:**
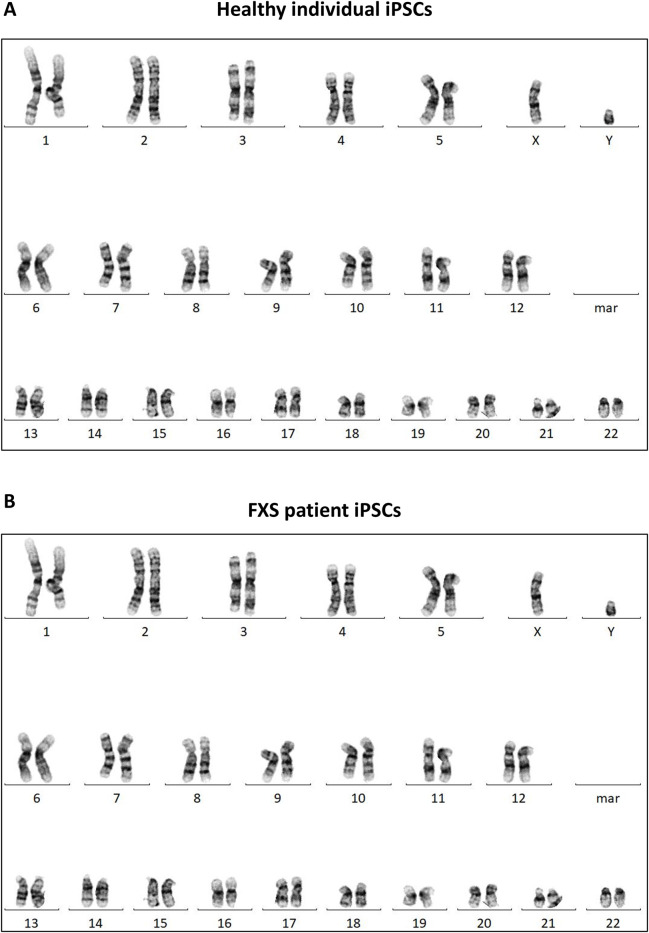
Karyotyping of the iPSC lines generated in this paper. Representative karyotype of a control iPSC line **(A)** and a FXS iPSC line **(B)**. Karyotypes for all cell lines can be found in Supplementary Figure S3.

Chromosomal alterations can occur during cell reprogramming so it is imperative to evaluate the genomic integrity of newly reprogrammed iPSC lines. An iPSC line should demonstrate a normal karyotype, devoid of any observable chromosomal abnormalities (Figure 6). Accordingly, all iPSC lines discussed in this paper demonstrated a normal karyotype which can be found in Supplementary Figure S3.

#### 5.3.3 Functional assessment of pluripotency

**FIGURE 7:**
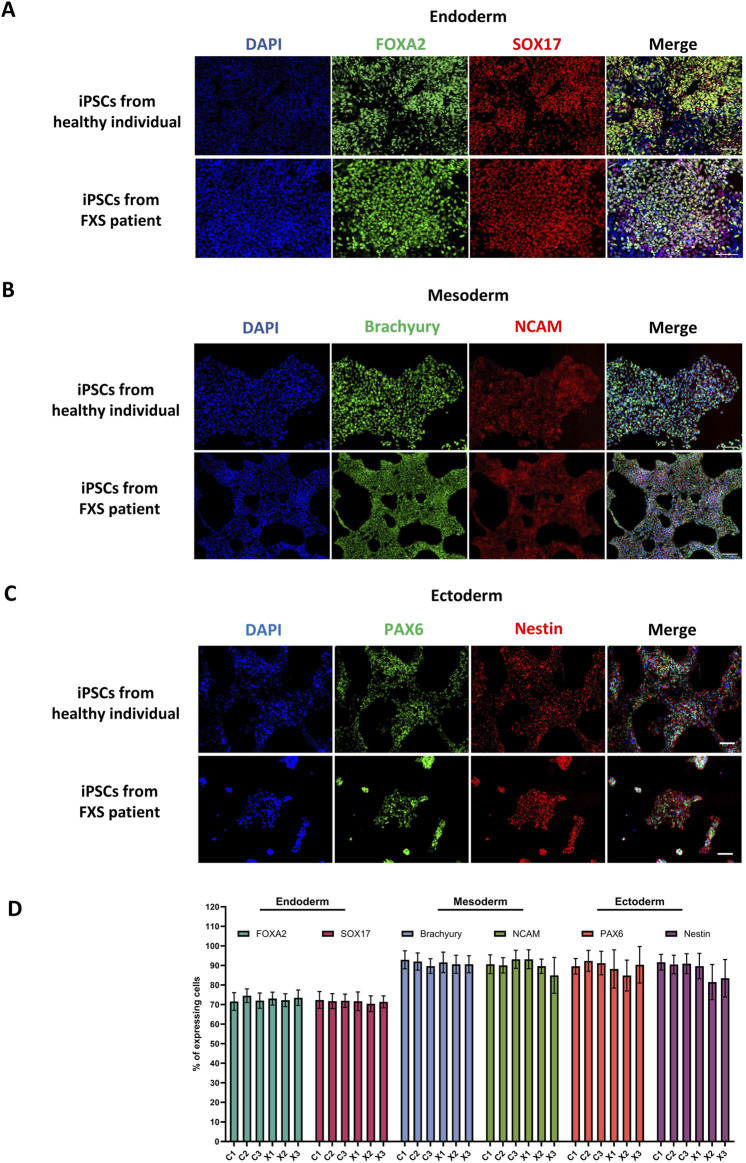
Functional assessment of pluripotency via trilineage differentiation. Representative immunostainings of the ectodermal markers FOXA2 and SOX17 **(A)**, mesodermal markers Brachyury and NCAM **(B)** and ectodermal markers PAX6 and Nestin **(C)** in a control and FXS iPSC lines. Immunostaining of all iPSCs lines produced in this paper can be found in Supplementary Figures S4–S6. Scale bar = 100 µm. **(D)** Quantification of lineage-specific markers expression in all iPSCs lines (n = 10 fields of views per cell line, mean ± SD).

A critical aspect of the validation of a newly reprogrammed iPSC line, is assessing its functional pluripotency. Thus, proper iPSCs should demonstrate the capacity to differentiate into all three germ lineages. The functional pluripotency assay used in this paper enables the swift differentiation of iPSCs into precursor cells of each germ lineage. Assessing differentiation efficiency involves monitoring the expression of specific markers for each lineage. Consequently, the majority of cells (˃60%) within the culture should exhibit differentiation to confirm the iPSC line’s pluripotency. All iPSCs lines described in this paper successfully showed differentiation into the three germ layers. A representative example of a control and FXS iPSC lines can be found in Figure 7. Results from all cell lines described in this paper can be found in Supplementary Figures S4–S6.

#### 5.3.4 Assessment of sterility and *mycoplasma* testing

All iPSC lines should be negative for bacterial, fungi and *mycoplasma* contamination, otherwise they could exhibit self-differentiation and/or other abnormal cell phenotypes. Contaminated cell lines should be discarded. If contamination is identified, cryopreserved cells from earlier passages should also be checked for contamination.

## 6 Discussion

Cells from skin, blood and urine samples are routinely used for the establishment of iPSC lines through cellular reprogramming. The reprogramming efficiency depends on the expression mode of OSKM factors (e.g., episomal vectors, Sendai virus, adenovirus) and the type of somatic cells. In this paper, we described a robust method to cultivate primary UDCs and efficiently reprogram them into iPSCs using a feeder-free and non-integrative system. This protocol is reliable, reproducible and was used to generate and validate iPSC lines from three healthy individuals and three FXS patients. To our knowledge, this is the first report describing the generation of iPSCs from UDCs of FXS patients.

The method described in this paper demonstrates several advantages over other reprogramming methodologies: 1) Urine offers a non-invasive supply of somatic cells that can be easily and repeatedly collected, extracted and cultivated without the need for medical personal. This is not the case for skin samples that requires a biopsy and can be quite invasive for young patients. 2) Reprogramming efficiency of UDCs is usually higher (0.1%–4%) than fibroblasts (0.01%-0.5%) and PBMCs (0.01%-0.05%) (Loh et al., 2009; Warren et al., 2010; Raab et al., 2014; Schlaeger et al., 2015; Kim et al., 2016). 3) The use of Sendai virus allows for efficient and non-integrative delivery of OSKM factors into UDCs. This overcomes the limitations of other commonly used reprogramming methods, such as the low efficiency of RNA or episomal vectors transfection and the random genomic integration following lentiviral infection (Rao and Malik, 2012; Cerneckis et al., 2024). 4) Using a feeder-free system offers greater convenience and reproducibility compared to feeder-dependent systems that require the co-culture fibroblasts layer to produce the extracellular matrix needed for iPSC culture (Liu et al., 2020; Wang et al., 2023). 5) The validation procedure outlined guarantee the quality of the newly generated iPSC lines, thereby ensuring the robustness of the research conducted with these cell lines (Ludwig et al., 2023).

However, despite its robustness and overall great efficiency, this reprogramming method has some pitfalls. First, we were only able to successfully establish a primary UDC culture from around 50% of the urine samples we processed (Supplementary Table S1), which is consistent with previous reports (Dörrenhaus et al., 2000; Wang et al., 2013; Li et al., 2014). The quantity and quality of urine samples are very variable and highly influence the rate with which UDC culture can be established. This rate can certainly be increased with further optimized culture conditions but also better sample collection and processing. For instance, urine samples collected the morning in the first 4 h after waking up tend to give the best UDC establishment. Another limitation is the prolonged duration of UDC culture prior to reprogramming, which exceeds the time typically required for the culture of fibroblasts and PBMCs (Li et al., 2014; Raab et al., 2014).

Alternative methodologies can be applied during the validation procedures outlined in this paper and should be selected based on user preference and available equipment. For instance, pluripotency marker expression can also be assessed using quantitative polymerase chain reaction (qPCR) or fluorescence-activated cell sorting (FACS) (Suresh Babu et al., 2023). Besides G-banding karyotyping, genomic integrity can be validated through methods such as fluorescent *in situ* hybridization (FISH) and comparative microarray techniques such as qPCR and digital droplet PCR. These techniques differ in term of genomic resolution, sensitivity, and cost, and should therefore be chosen according to the user’s requirements. (Assou et al., 2018). Finally, functional assessment of pluripotency can be performed using other differentiation protocols and markers than those outlined above (Baghbaderani et al., 2016; Rao and Malik, 2012).

UDCs offer a unique source of patient-specific pluripotent cells that facilitates the development of drug screening and tailored therapeutic approaches. Importantly, UDC-derived iPSCs have demonstrated strong efficacy in disease modeling, allowing researchers to recapitulate patient-specific pathologies *in vitro* and enhance our understanding of disease mechanisms (Zhang et al., 2015; Cao et al., 2018; Ghori and Wahid, 2021; Supakul et al., 2022; Baliña-Sánchez et al., 2023; Park et al., 2023; Tian and Yin, 2023; Yin et al., 2024). Despite several challenges and considerations (i.e., standardization of reprogramming protocols, genomic stability), urine-derived iPSCs serves as a highly versatile resource in the field of NDDs research, where they hold hopeful promise for precision and regenerative medicine.

## Data Availability

The original contributions presented in the study are included in the article/supplementary material, further inquiries can be directed to the corresponding authors.
