# Supplementary material for: Efficient generation of human induced pluripotent stem cells from urine samples of patients with Fragile X syndrome
Source: Front Cell Dev Biol. 2024 Nov 22;12:1489190. doi: 10.3389/fcell.2024.1489190 (PMC11621072; doi:10.3389/fcell.2024.1489190)
Supplement: Supplementary file 1 [file DataSheet1.pdf]

Supplementary figure legends

Supplementary Figure 1: Freshly extracted urine samples are highly heterogenous, containing a diverse array of both biological and non-biological constituents. (**A-B)** Urine samples from female donors contain an important number of cells mainly consisting of squamous cells that do not adhere to the culture vessels. **(C)** Urine sample from a male donor containing a few numbers of spherical cells. **(D)** Non-biological objects might also be present in some urine cultures.

Supplementary Figure 2: Representative immunostaining of pluripotency markers: **(A)** Nanog, **(B)** OCT 4, **(C)** SOX2 and **(D)** FMRP in all iPSC lines produced in this paper. Scale bar = 100 µm.

Supplementary Figure 3: Karyotype for all iPSC lines produced in this paper.

Supplementary Figure 4: Representative immunostaining for the endoderm markers FOXA2 and SOX17 following the functional pluripotency assay. Scale bar = 100 µm.

Supplementary Figure 5: Representative immunostaining for the mesoderm markers Brachyury and NCAM following the functional pluripotency assay. Scale bar = 100 µm.

Supplementary Figure 6: Representative immunostaining for the ectoderm markers Pax6 and Nestin following the functional pluripotency assay. Scale bar = 100 µm.

Supplemental Figure 1

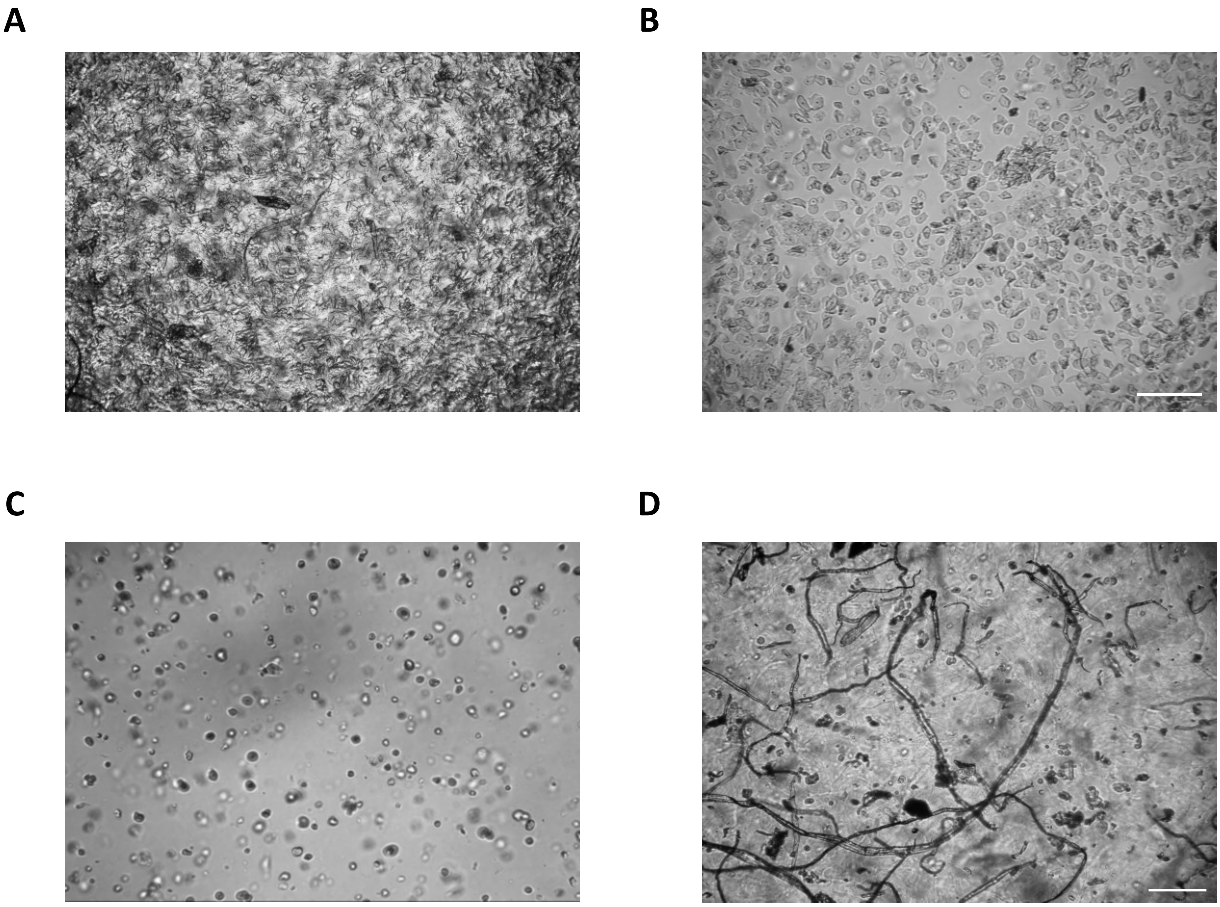

Supplemental Figure 2

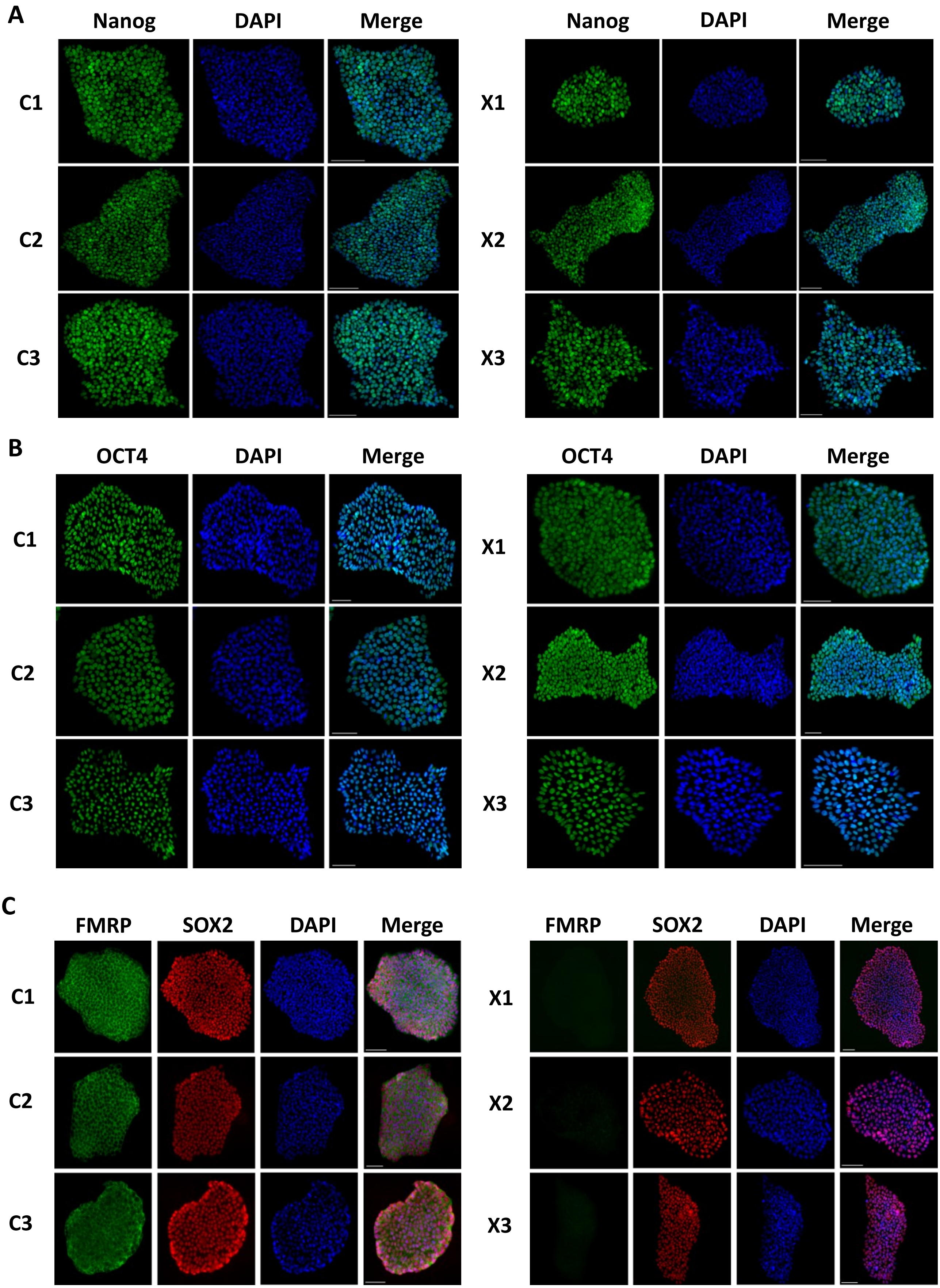

Supplemental Figure 3

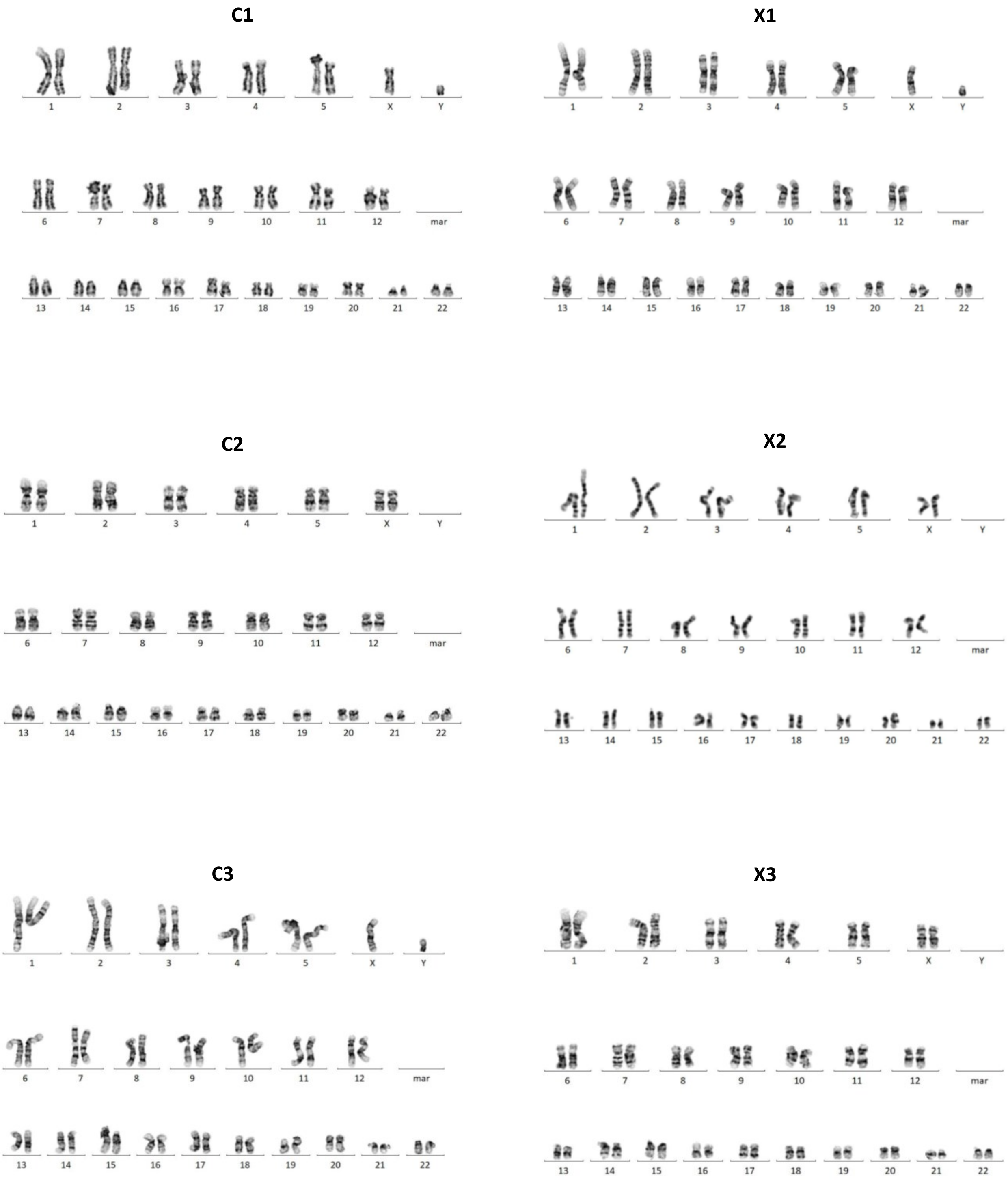

Supplemental Figure 4

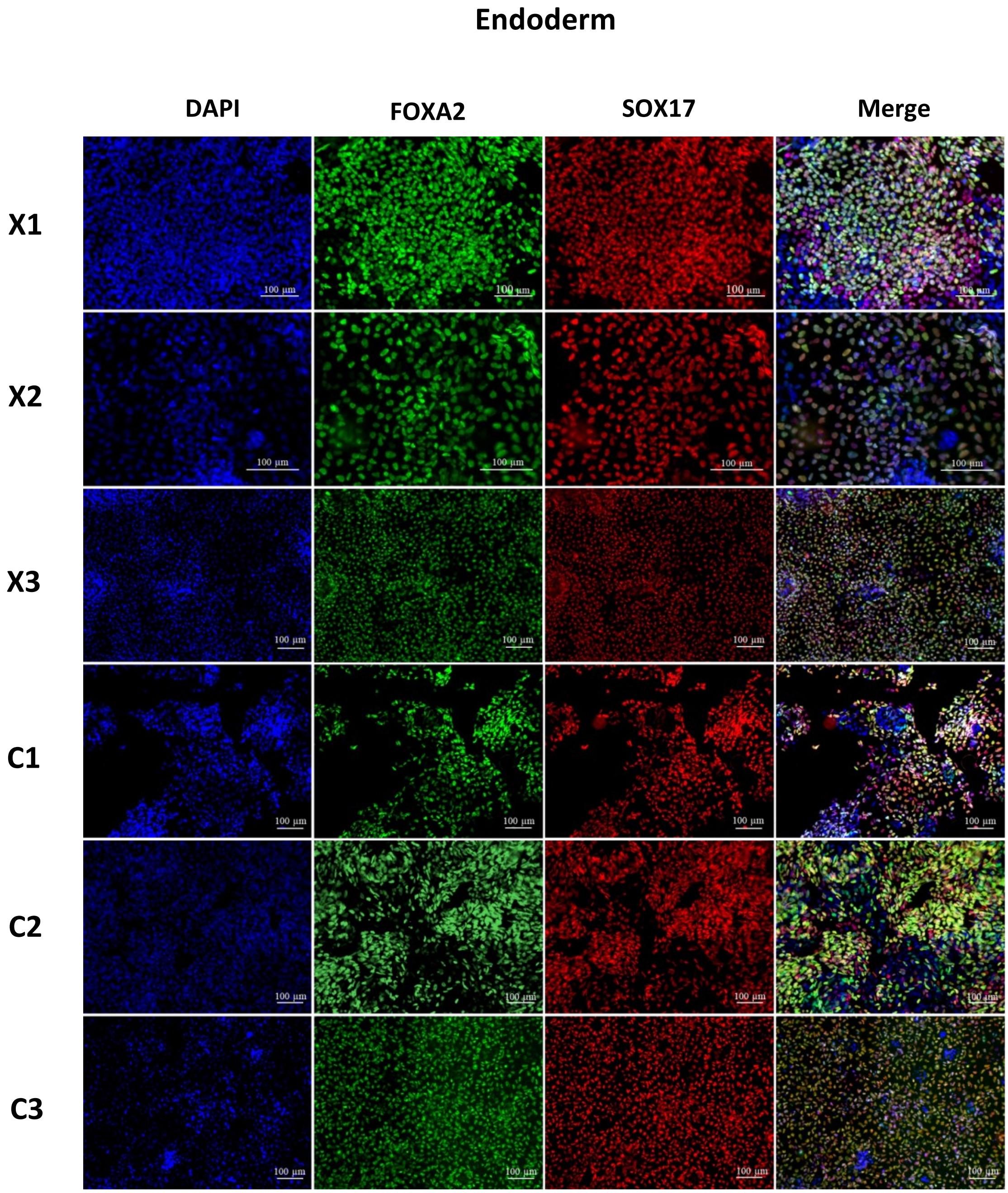

Supplemental Figure 5

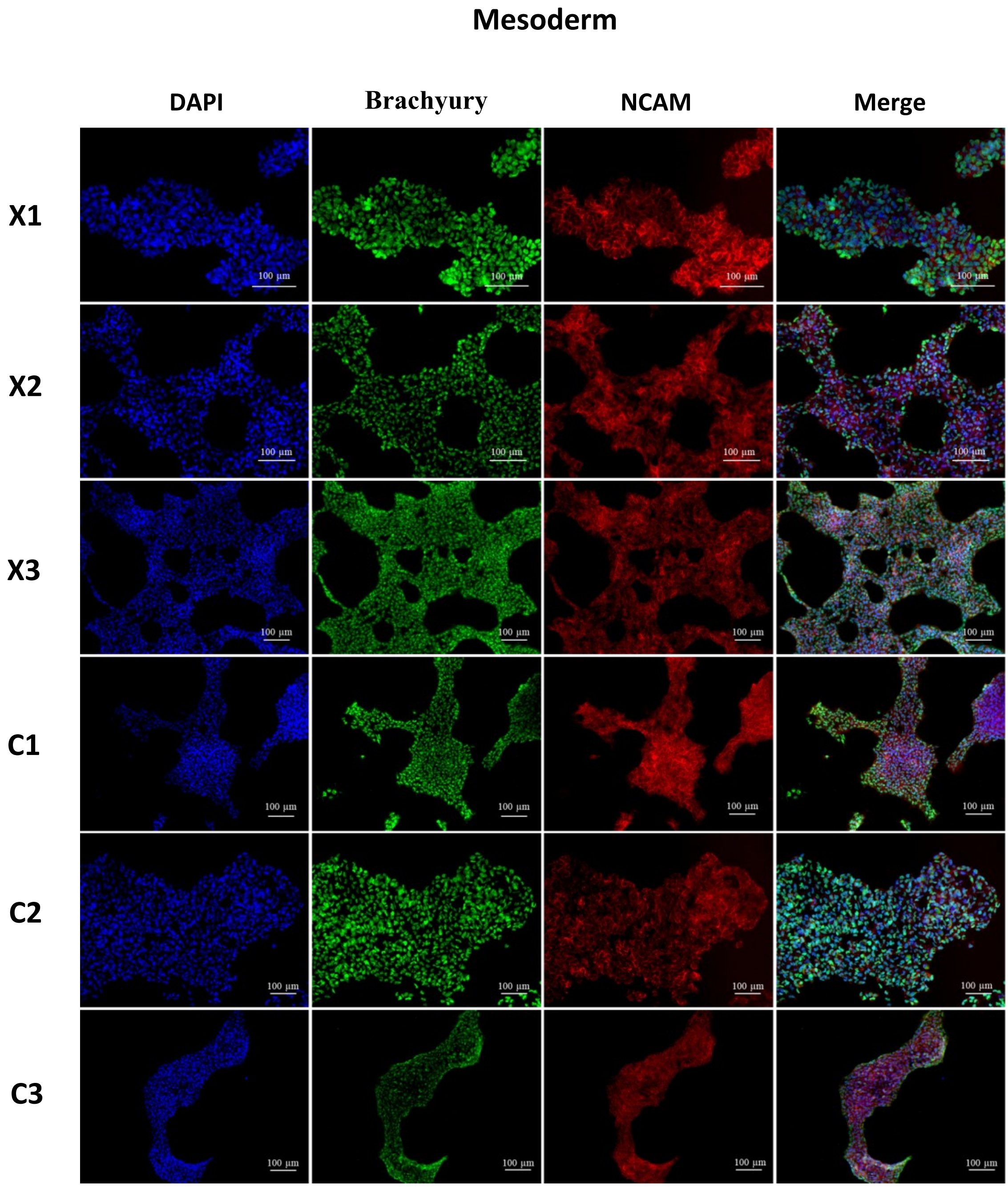

Supplemental Figure 6

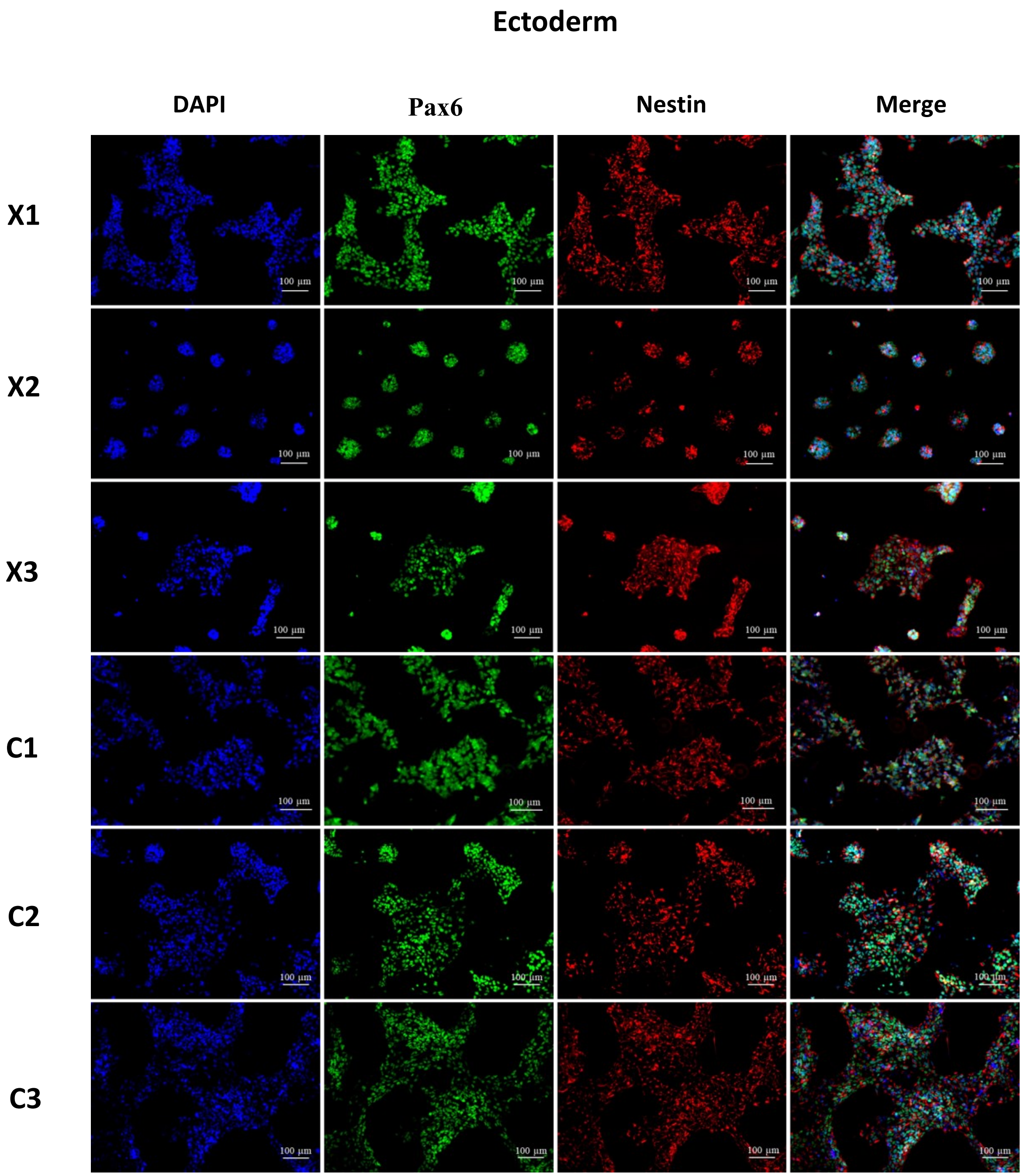

Supplementary Table 1: Efficiency of UDC culture establishment

Genotype	Number of urine samples collected and treated (total)	Number of urine samples collected and treated (male)	Number of urine samples collected and treated (female)	Number of UDCs culture successfully established (total)	Number of UDCs culture successfully established (male)	Number of UDCS culture successfully established (female)
CTL	15	10	5	7 (53%)	4 (40%)	3 (60%)
FXS	7	3	4	3 (43%)	1 (33%)	2 (50%)
